# The impact of similarity metrics on cell-type clustering in highly multiplexed in situ imaging cytometry data

**DOI:** 10.1093/bioadv/vbad141

**Published:** 2023-10-09

**Authors:** Elijah Willie, Pengyi Yang, Ellis Patrick

**Affiliations:** Sydney Precision Data Science Centre, The University of Sydney, Camperdown, NSW 2006, Australia; School of Mathematics and Statistics, The University of Sydney, Camperdown, NSW 2006, Australia; Sydney Precision Data Science Centre, The University of Sydney, Camperdown, NSW 2006, Australia; School of Mathematics and Statistics, The University of Sydney, Camperdown, NSW 2006, Australia; Laboratory of Data Discovery for Health Limited (D24H), Science Park, Hong Kong, China; Computational Systems Biology Group, Children’s Medical Research Institute, The University of Sydney, Westmead, NSW 2145, Australia; Sydney Precision Data Science Centre, The University of Sydney, Camperdown, NSW 2006, Australia; School of Mathematics and Statistics, The University of Sydney, Camperdown, NSW 2006, Australia; Laboratory of Data Discovery for Health Limited (D24H), Science Park, Hong Kong, China; Centre for Cancer Research, The Westmead Institute for Medical Research, The University of Sydney, Westmead, NSW 2145, Australia

## Abstract

**Motivation:**

The advent of highly multiplexed in situ imaging cytometry assays has revolutionized the study of cellular systems, offering unparalleled detail in observing cellular activities and characteristics. These assays provide comprehensive insights by concurrently profiling the spatial distribution and molecular features of numerous cells. In navigating this complex data landscape, unsupervised machine learning techniques, particularly clustering algorithms, have become essential tools. They enable the identification and categorization of cell types and subsets based on their molecular characteristics. Despite their widespread adoption, most clustering algorithms in use were initially developed for cell suspension technologies, leading to a potential mismatch in application. There is a critical gap in the systematic evaluation of these methods, particularly in determining the properties that make them optimal for in situ imaging assays. Addressing this gap is vital for ensuring accurate, reliable analyses and fostering advancements in cellular biology research.

**Results:**

In our extensive investigation, we evaluated a range of similarity metrics, which are crucial in determining the relationships between cells during the clustering process. Our findings reveal substantial variations in clustering performance, contingent on the similarity metric employed. These variations underscore the importance of selecting appropriate metrics to ensure accurate cell type and subset identification. In response to these challenges, we introduce FuseSOM, a novel ensemble clustering algorithm that integrates hierarchical multiview learning of similarity metrics with self-organizing maps. Through a rigorous stratified subsampling analysis framework, we demonstrate that FuseSOM outperforms existing best-practice clustering methods specifically tailored for in situ imaging cytometry data. Our work not only provides critical insights into the performance of clustering algorithms in this novel context but also offers a robust solution, paving the way for more accurate and reliable in situ imaging cytometry data analysis.

**Availability and implementation:**

The FuseSOM R package is available on Bioconductor and is available under the GPL-3 license. All the codes for the analysis performed can be found at Github.

## 1 Introduction

Technological advancements over the past decade have provided researchers the capability to simultaneously measure multiple molecular features in tissue at subcellular resolution (Lewis *et al.* 2021). Key technologies that are pioneering a new era for spatially resolved proteomics include imaging mass cytometry (IMC) (Giesen *et al.* 2014), multiplexed ion beam imaging by time of flight (MIBI-TOF) (Keren *et al.* 2019), co-Detection by indEXing (CODEX) (Black *et al.* 2021), and its successor phenocycler. These technologies can measure ∼50–100 features with high throughput, enabling researchers to address complex questions about the spatial distribution and interaction of various types of cells *in situ* (Baharlou *et al.* 2019). A ubiquitous analytical step when analyzing highly multiplexed imaging data is defining functionally distinct cell groupings. While there have been recent developments in spatial analysis approaches that simultaneously phenotype cells by their cellular environment and molecular features (Lee *et al.* 2023, Liu *et al.* 2023), the most commonly used phenotyping approaches only use molecular features and are not intentionally biased by cellular interactions.

Unsupervised clustering algorithms are valuable tools for discovering both known and novel cell types in highly multiplexed data, even in cases where prior knowledge of the cell types present in an experiment is lacking (Karim *et al.* 2021). Here, we use the terminology “cell type” liberally, with clusters also potentially representing distinct known or novel cell states. In our review of the literature, over 70% of manuscripts employing highly multiplexed imaging data for analysis utilized one of three clustering algorithms. IMC and MIBI-TOF data were predominately clustered using either Phenograph (Levine *et al.* 2015), a graph-based Louvain community detection method, or FlowSOM (Van Gassen *et al.* 2015), a self-organizing map (SOM) approach, while CODEX data was predominately clustered using X-shift, a *k*-nearest neighbor (KNN) algorithm accessible through the Vortex GUI (Samusik *et al.* 2016). In the remaining manuscripts, other Louvain and Leiden graph-based community detection algorithms, hierarchical clustering, and K-means clustering were used.

Despite their popularity in imaging modalities, Phenograph, FlowSOM, and X-shift were developed in 2015 and 2016 for suspension cytometry technologies, which do not share all of the same technical limitations and noise profiles with tissue-based imaging technologies. Technical artifacts present in most imaging technologies include non-specific binding (Batth *et al.* 2020) and lateral marker spillover (Bai *et al.* 2021). Additionally, in practice, these methods are often employed to generate a large set of candidate clusters, which using expert domain knowledge are then manually clustered, refined, and annotated based on biological features, such as key marker expression, and cell localization. Following this, there exist multiple avenues for further exploration of how clustering algorithms could be tailored for multiplexed imaging data.

Choosing an appropriate similarity metric is crucial for clustering algorithms as it determines how points in a dataset, in our case cells, are partitioned into clusters. Different similarity metrics, often referred to as distance metrics, can yield different clusters. While the Euclidean distance is commonly used in many clustering algorithms, recent studies have shown that correlation-based metrics, such as Pearson or Spearman correlation perform better when clustering in other multiplexed single-cell technologies (Kim *et al.* 2019, Watson *et al.* 2022). Evaluating the performance of different similarity metrics for defining cell types in multiplexed imaging data may guide the improvement or development of new clustering algorithms, which are optimal for these exciting technologies.

In this study, we systematically assess the performance of various distance- and correlation-based metrics in 15 imaging datasets, using multiple performance metrics, such as the Adjusted Rand Index (ARI), the Normalized Mutual Information (NMI), the F-Measure, and the Fowlkes–Mallows Index (FM-Index). We also compare the performance of best-practice clustering methods that currently employ different similarity metrics. Based on our assessment, which highlights the benefits of combining information from multiple similarity metrics, we introduce a new clustering algorithm called FuseSOM. FuseSOM utilizes SOMs and combines multiple similarity metrics through multiview ensemble learning and hierarchical clustering. This algorithm aims to accurately and robustly identify cell types in multiplexed *in situ* imaging cytometry assays. Overall, our work demonstrates the impact of similarity metrics on clustering cells in multiplexed imaging cytometry data and proposes FuseSOM as a promising method for the analysis of such data.

## 2 Methods

### 2.1 Datasets

To benchmark the performance of FuseSOM on imaging datasets from various technologies, we curated a set of 15 datasets generated using different imaging technologies. We selected datasets with human intervention in manually gating cell populations or merging biologically similar clusters. The intention of selecting datasets with manual intervention in defining cell types is to reduce bias toward the original clustering method when evaluating clustering performance. Using these types of datasets also provides higher confidence in the quality of clusters since expert domain knowledge has been applied to scrutinize the clusters further. Datasets were sourced from major databases, including Zenodo, Figshare, and Mendeley. When available, we used the version data that had been processed as described in the original manuscript. We also used the same markers and the same final number of clusters for clustering as described in the manuscript. The imaging technologies used included CODEX (Black *et al.* 2021) (four datasets), IMC (Giesen *et al*. 2014) (six datasets), MIBI-TOF (Keren *et al.* 2019) (four datasets), and sequential Fluorescence *In Situ* Hybridization (seqFISH) (one dataset) (Eng *et al.* 2019). See Table 1 for a detailed description of the datasets used.

### 2.2 Evaluation metrics

To evaluate clustering performance for clustering solutions generated across methods, we used a set of methods; the ARI, NMI, FM-Index, and the F-Measure (Fowlkes and Mallows 1983, Steinley 2004, Hripcsak and Rothschild 2005, Kvålseth 2017). The ARI measures the similarity between two data clusterings, adjusting for chance


(1) $$\mathrm{ARI}=\frac{\sum_{ij}\left(\begin{array}{c} n_{ij}\\ 2 \end{array}\right)-\left[\sum_{i}\left(\begin{array}{c} a_{i}\\ 2 \end{array}\right)\sum_{j}\left(\begin{array}{c} b_{j}\\ 2 \end{array}\right)\right]/\left(\begin{array}{c} n\\ 2 \end{array}\right)}{\frac{1}{2}\left[\sum_{i}\left(\begin{array}{c} a_{i}\\ 2 \end{array}\right)+\sum_{j}\left(\begin{array}{c} b_{j}\\ 2 \end{array}\right)\right]-\left[\sum_{i}\left(\begin{array}{c} a_{i}\\ 2 \end{array}\right)\sum_{j}\left(\begin{array}{c} b_{j}\\ 2 \end{array}\right)\right]/\left(\begin{array}{c} n\\ 2 \end{array}\right)},$$

where $n_{ij}$ is the number of pairs of elements that are in the same set in both clusterings, $a_{i}$ is the total number of pairs in the same set for the first clustering, $b_{j}$ is the total number of pairs in the same set for the second clustering, and *n* is the total number of elements.

NMI is a normalization of the mutual information (MI) score to scale the results between zero (no MI) and one (perfect correlation) and is defined as follows:


(2) $$\mathrm{NMI}(X,Y)=\frac{2\times I(X,Y)}{H(X)+H(Y)},$$

where I(X,Y) is the MI between clusters *X* and *Y*, and H(X)H(Y) are the entropies of clusters *X* and *Y*, respectively.

The FM-Index is the geometric mean of precision and recall, and it is defined as follows,


(3) $$\mathrm{FM}=\sqrt{\frac{\mathrm{TP}}{\mathrm{TP}+\mathrm{FP}}\times \frac{\mathrm{TP}}{\mathrm{TP}+\mathrm{FN}}},$$

where TP is the number of true positives, FP is the number of false positives, and FN is the number of false negatives.

The F-Measure, which is the harmonic mean of the precision and recall, is defined as follows:


(4) $$F=\frac{2\times \mathrm{Precision}\times \mathrm{Recall}}{\mathrm{Precision}+\mathrm{Recall}},$$

where


(5) $$\mathrm{Precision}=\frac{\mathrm{TP}}{\mathrm{TP}+\mathrm{FP}}$$

and


(6) $$\mathrm{Recall}=\frac{\mathrm{TP}}{\mathrm{TP}+\mathrm{FN}}.$$

All these metrics take values between zero and one, with zero being no similarity and one being perfect similarity.

### 2.3 Distance metrics

Six types of metrics across two classes that are predominantly used across machine-learning clustering literature were used in this study. The two classes include correlation-based and distance-based. The distance-based metrics were Euclidean, Manhattan, and Maximum distance, while the correlation-based metrics included Pearson correlation, Spearman correlation, and Cosine similarity. More formally, let $x_{im}$ and $x_{jm}$ denote the expression of a marker m=1,…,M in cell i=1,…,N and cell j=1,…,N, where *G* and *N* are the total number of markers and cells, respectively. Let $D=d_{ij}$ be a distance matrix, where $d_{ij}$ represents the distance between cell_i_ and cell_j_. We can then define the distance-based metrics as follows:

Euclidean distance,


(7) $$d_{ij}=\sqrt{\sum_{m=1}^{M}{\left(x_{im}-x_{jm}\right)}^{2}},$$

Manhattan distance,


(8) $$d_{ij}=\sum_{m=1}^{M}|x_{im}-x_{jm}|,$$

Maximum distance,


(9) $$d_{ij}=\underset{m}{\max}|x_{im}-x_{jm}|.$$

Similarly, the correlation-based metrics can be defined as follows:

Pearson distance,


(10) $$d_{ij}=\sqrt{2\left(1-\frac{\sum_{m=1}^{M}\left(x_{im}-{\bar{x}}_{i}\right)(x_{jm}-{\bar{x}}_{j})}{\sqrt{\sum_{m=1}^{M}{\left(x_{im}-{\bar{x}}_{i}\right)}^{2}}\sqrt{\sum_{m=1}^{M}{\left(x_{jm}-{\bar{x}}_{j}\right)}^{2}}}\right)},$$

Spearman distance,


(11) $$d_{ij}=\sqrt{2\left(1-\frac{\sum_{m=1}^{M}\left(r_{im}-{\bar{r}}_{i}\right)(r_{jm}-{\bar{r}}_{j})}{\sqrt{\sum_{m=1}^{M}{\left(r_{im}-{\bar{r}}_{i}\right)}^{2}}\sqrt{\sum_{m=1}^{M}{\left(r_{jm}-{\bar{r}}_{j}\right)}^{2}}}\right)},$$

Cosine distance,


(12) $$d_{ij}=\sqrt{2\left(1-\frac{\sum_{m=1}^{M}x_{im}x_{jm}}{\sqrt{\sum_{m=1}^{M}x_{im}^{2}}\sqrt{\sum_{m=1}^{M}x_{jm}^{2}}}\right)},$$

where $r_{ij}$ is the rank of marker *m* in $\mathrm{cel}l_{i}$, $\bar{x_{i}}$ is the mean expression of cell_i_, $\bar{x_{j}}$ is the mean expression of cell_j_, $\bar{r_{i}}$ is the mean expression rank of cell_i_, and $\bar{r_{j}}$ is the mean expression rank of cell_j_.

### 2.4 Clustering algorithms

For this work, a few clustering algorithms were used for comparing the effects of distance metrics on clustering outcomes. These algorithms include hierarchical clustering, FlowSOM (Van Gassen *et al.* 2015), K-means, and Phenograph (Levine *et al.* 2015).

The “hierarchical clustering” builds a hierarchy of clusters by either merging smaller clusters into larger ones (agglomerative) or dividing a large cluster into smaller ones (divisive) using a linkage function. The process continues iteratively, resulting in a tree-like diagram called a dendrogram that represents the nested clusters. The agglomerative version was used with the average linkage function (Nielsen 2016).

The “FlowSOM” utilizes SOMs and hierarchical clustering to analyze and visualize complex datasets, particularly in flow cytometry. It groups cells into nodes on a grid based on similarity, providing insights into data structures. This technique is especially valuable in identifying and understanding cell populations. The FlowSOM algorithm (version 2.8.0) was obtained from Bioconductor.

The “K-means” clustering partitions data into “*k*” clusters by repeatedly assigning data points to the nearest centroid and recalculating the centroids. The process continues iteratively until the centroids stabilize. The “Base R” implementation of the K-means algorithm was used.

The “Phenograph” is a clustering method that constructs a KNN graph from data, usually applied to single-cell data analysis. Community detection is performed on this graph using the Louvain method to identify clusters or communities of similar nodes. The “phenograph” function from the *ReductionWrappers* version 2.5.4 (Smith 2023) R package was used.

### 2.5 FuseSOM

Here, the FuseSOM algorithm is described. The algorithm starts by taking in an *m* by *n* matrix, where *m* is the number of cells and *n* is the number of markers. Next, FuseSOM uses this matrix to generate a SOM. A SOM is a type of dimensionality reduction algorithm that maps points in a high dimensional space (d>2) to a lower dimensional space (d=2). The SOM architecture preserves the topological relationships between points when reduced to a lower dimension. The SOM also provides a set of points called prototypes, which are representations of points in the higher dimensional space. The SOM architecture was chosen due to its ability to preserve topological structures of the input data, and its ability to represent complex non-linear relationships in the data, allowing them to capture more intricate patterns and dependencies. Like cluster centers in the k-means algorithm, many points can be mapped to a single prototype. For a more thorough treatment of SOMs, see Miljkovic (2017). The *YASOMI* package (version 0.3) was obtained and modified to implement the SOM used in FuseSOM (Rossi 2012).

In this work, we generate a SOM, and the prototypes are used for clustering. After clustering, the clusters are projected back to the original data to classify the original data points. The SOM algorithm requires a 2-*d* grid(*x*, *y*) size, which determines the number of prototypes. Grid sizes of varying shapes are allowed. However, square grids are typically used. To estimate the size of the grid for a dataset, we use the method described in Patterson *et al.* (2006). This method computes the number of eigenvalues of a covariance matrix significantly different from the Tracy–Widom distribution (Tracy and Widom 1994).

Next, multiview integration combines the Pearson correlation distance, Cosine distance, Spearman correlation, and Euclidean distance between the prototypes to generate a final distance matrix for clustering. Multiview ensemble learning is a machine-learning strategy that employs multiple diverse views or perspectives of the same data to enhance predictive modeling. In a typical multiview ensemble learning setup, each view represents a unique set of features or a unique preprocessing or transformation of the data. These different views capture various aspects of the data, which when combined, offer a more comprehensive and potentially more accurate representation. In this work, the different views are represented by the various distances computed between the cells. To combine these views, we adopted a multiview integration (Fuse) method to combine the four transformed matrices (Melssen *et al.* 2006). Formally, the multiview integration can be defined as follows:


(13) $$D_{\mathrm{fused}}[i,k]=\sum w_{i}\times D[\mathrm{ijk}],$$

where $D_{\mathrm{fused}}[i,j]$ is the combined dissimilarity between samples *j* and *k*, $w_{i}$ is the weight assigned to the ith dissimilarity matrix, and *D*[*ijk*] is the dissimilarity between *j* and *k* for the ith dissimilarity matrix. All distances are weighted equally. We tried a variety of weighting methods and the equal weighting (Fuse) consistently performed the best. See Supplementary Fig. S5.

The *analogue* package (version 0.17-6) is used to perform the multiview integration (Simpson and Oksanen 2021). The *psych* package (version 2.3.3) transforms the similarity matrices into distance matrices (Revelle 2022). Correlations and cosines are transformed into distances by using the formula:


(14) $$D=\sqrt{2(1-r_{ij})},$$

where $r_{ij}$ is the value of the correlation or cosine between feature *i* and *j*. Finally, to generate final cluster labels using the integrated distance matrices, FuseSOM takes in a parameter *k*, which is the number of desired clusters. Next, hierarchical clustering using the average linkage function is used to generate the final clustering solution. The *FCPS* package (version 1.3.1) was used for hierarchical clustering (Thrun and Stier 2021).

### 2.6 Clustering framework

For consistency when comparing distance metrics across various datasets, each dataset was sampled five times to obtain 20K cells. After this, we executed each clustering algorithm and recorded the scores based on different evaluation metrics. To compare with FuseSOM, we used a substratification framework for each dataset. This framework accepts a dataset and produces five stratified samples. The purpose of substratification is to account for potential variability in clustering outcomes. Through this framework, when a dataset is inputted, it yields five stratified datasets. Stratification involves selecting 50% of cells from every annotated class (Kim *et al.* 2019).

### 2.7 Cluster size estimation

For most clustering algorithms, the number of clusters *k* is an important hyperparameter that must be set. To this end, many methods have been developed to help practitioners choose an appropriate number for their dataset. We have included well-known methods for estimating the number of clusters as part of the FuseSOM package. These methods include the Gap statistic, the Slope statistic, the Jump statistic, the Silhouette statistic, and the within-cluster distance (WCD) (Rousseeuw 1987, Tibshirani *et al.* 2001, Sugar and James 2003, Fujita *et al.* 2014).

The “Gap statistic” compares the change in the within-cluster sum of squares (WSS) from the observed data to that of a random clustering. A large gap value indicates the observed data has a more pronounced clustering structure than expected under a random scenario.

The “Silhouette statistic” quantifies how close each data point in one cluster is to data points in neighboring clusters, with values ranging from −1 to 1; higher values indicate better-defined clusters.

The “Jump statistic” evaluates the rate of increase in the WSS as a function of the number of clusters, with large jumps indicating the possible presence of distinct groups.

The “slope statistic” identifies an “elbow” or bend in the WSS plot; the point before the stabilization or decline in the slope can suggest an optimal number of clusters.

The “WCD” measures the compactness of clusters. A smaller value indicates tighter, more well-defined clusters. Each of these statistics offers unique insights and their combined interpretation aids in selecting an appropriate number of clusters.

We also implemented a “Discriminant” method for estimating the number of clusters based on the projection pursuit of the discriminant maximum clusterability. To accomplish this, we couple hierarchical clustering with discriminant analysis and multimodality testing to estimate the number of clusters (Silverman 1981, Hartigan and Hartigan 1985, Etemad and Chellappa 1997, Samadani *et al.* 2013, Mokari *et al.* 2018, Ameijeiras-Alonso *et al.* 2019). First, we generate a dendrogram using hierarchical clustering with average linkage. Next, for each node in the resulting tree, we project the two classes onto a line such that both classes are well separated. See Supplementary Fig. S6. The dip test for multimodality testing is then applied to the distribution of the points along this line (Hartigan and Hartigan 1985). The family-wise error rate is controlled using the method described in Meinshausen (2008). Finally, the number of nodes with significant *P*-values is returned as the number of clusters.

## 3 Results

### 3.1 Evaluating the impact of similarity metrics

**Figure 1.**
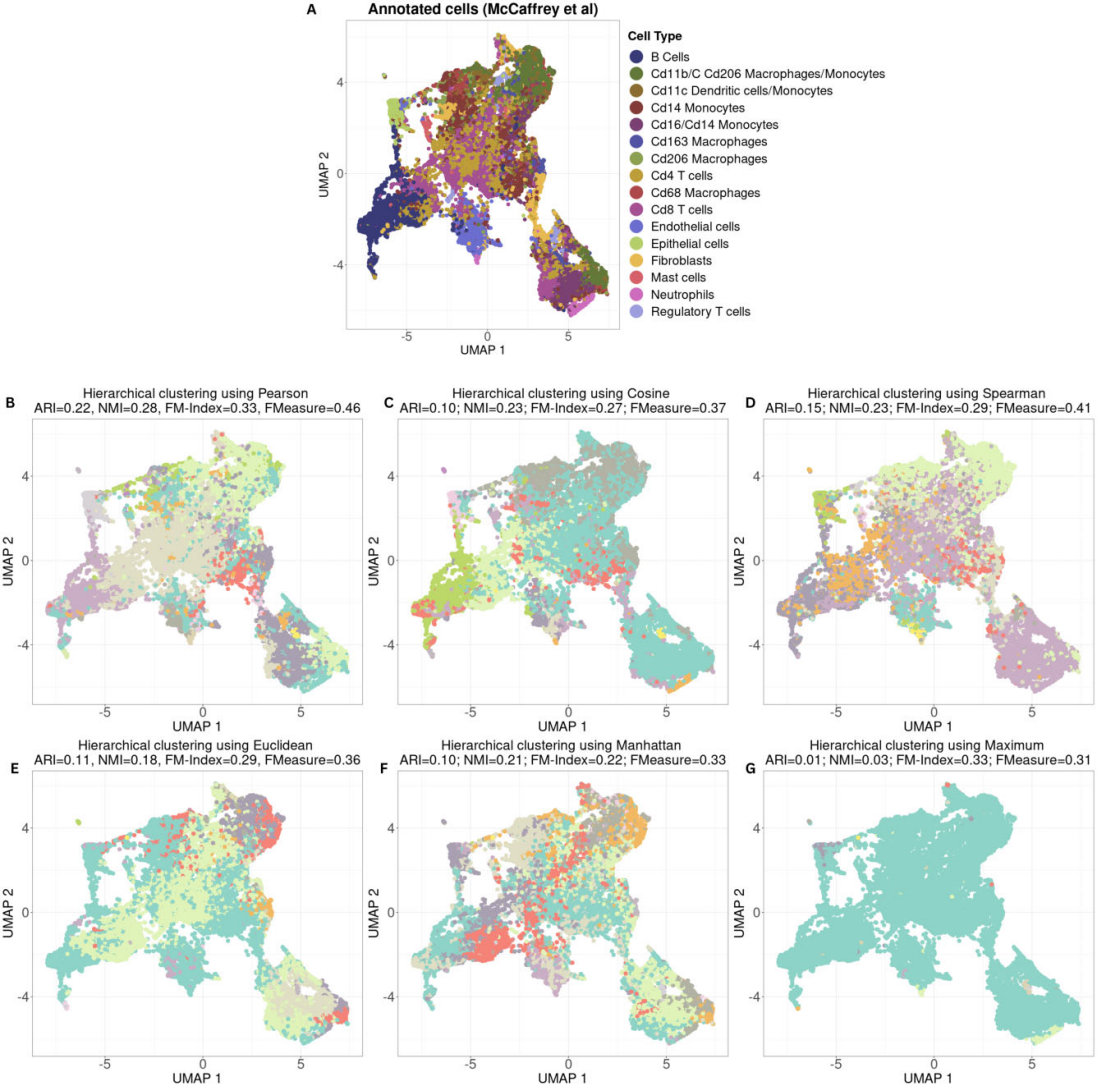
UMAP visualization of cells from a sample imaging dataset. (A) Cells colored by annotations from original study (McCaffrey *et al.* 2022). (B) Hierarchical clustering using Pearson’s correlation and concordance quantified by ARI, NMI, FM-Index, and F-Measure. (C) Hierarchical clustering using Cosine’s distance. (D) Hierarchical clustering using Spearman’s correlation. (E) Hierarchical clustering using Euclidean distance. (F) Hierarchical clustering using Manhattan distance. (G) Hierarchical clustering using Maximum distance.

To assess the impact of similarity metrics on clustering performance, we performed hierarchical clustering on a MIBI-TOF dataset using correlation-based metrics (Pearson, Spearman, and Cosine) or distance-based metrics (Euclidean, Manhattan, and Maximum) (McCaffrey *et al.* 2022). To assess performance, we compared the hierarchical clusters with the manually curated cell-type labels identified in the manuscript (Fig. 1). On average, correlation-based metrics outperform distance-based metrics by 8.0% for ARI, 10.7% for NMI, 1.70% for FM-Index, and 8.0% for F-Measure.

**Figure 2.**
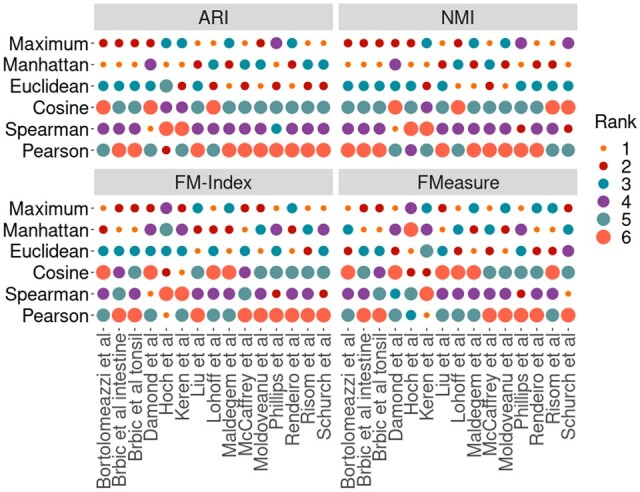
Benchmarking similarity metrics on agglomerative clustering of 15 multiplexed imaging datasets. Each dataset was subsetted to 20K cells five times, and the average clustering score was recorded. Results were ranked in descending order across all similarity metrics and datasets by each evaluation metric. A larger circle size indicates better performance. Correlation-based metrics are consistently ranked higher than distance-based metrics across most datasets.

To provide a comprehensive assessment of the performance of similarity metrics, we quantified the clustering performance of the metrics on 15 multiplexed *in situ* imaging cytometry datasets (Table 1). These datasets were chosen as each had some manual intervention when cell-type labels were defined. Each dataset was randomly subsampled to 20K cells five times, and each subset was clustered using hierarchical clustering with all the similarity metrics. Finally, the average was taken across the five subsets. Across the 15 datasets, correlation-based metrics consistently outperformed distance-based metrics (Fig. 2 and Supplementary Fig. S1), more accurately recapitulating the manually curated cell-type labels from their original publications. These results show the efficacy of correlation-based metrics in hierarchical clustering.

**Table 1.** Imaging datasets used.

Dataset	Technology	Num. markers	Num. cells	Num. celltypes	Disease	Tissue	Cell annotation method
Schürch *et al.* (2020)	CODEX	49	258 385	29	Colorectal cancer	Colon	X-shift (Samusik *et al.* 2016) then supervised cluster merging
Phillips *et al.* (2021)	CODEX	52	117 170	21	Lymphoma	Skin	X-shift (Samusik *et al.* 2016) then supervised cluster merging
Brbić *et al.* (2022)	CODEX	48	248 285	21	None	Small intestine/colon	Manually annotated
Brbić *et al.* (2022)	CODEX	44	219 926	13	Normal/Bartett’s esophagus	Tonsil	Manually annotated
Moldoveanu *et al.* (2022)	IMC	12	227 592	10	Melanoma	Skin	PhenoGraph (Levine *et al.* 2015) followed by K-means
Van Maldegem *et al.* (2021)	IMC	17	282 837	16	Lung cancer	Lung	PhenoGraph followed by manual splitting
Hoch *et al.* (2022)	IMC	41	864 263	10	Melanoma	Skin	Manual gating
Rendeiro *et al.* (2021)	IMC	38	515 791	17	Covid-19	Lung	Leiden followed by manual merging
Damond *et al.* (2019)	IMC	36	252 059	16	Type 1 diabetes	Pancreas	Supervised cell classifier
Bortolomeazzi *et al.* (2021)	IMC	30	218 615	9	Colorectal cancer	Colon	Seurat followed by DBSCAN
Risom *et al.* (2022)	MIBI-TOF	22	69 672	23	Breast cancer	Breast	FlowSOM (Van Gassen *et al.* 2015) followed by manual merging
Keren *et al.* (2019)	MIBI-TOF	16	201 656	6	TN breast cancer	Various	FlowSOM (Van Gassen *et al.* 2015) followed by merging by hierarchical merging
McCaffrey *et al.* (2022)	MIBI-TOF	37	30 943	16	Tuberculosis	Various	Iterative FlowSOM (Van Gassen *et al.* 2015) clustering
Liu *et al.* (2022)	MIBI-TOF	12	345 490	8	Various cancers	Various	FlowSOM (Van Gassen *et al.* 2015) followed by merging by hierarchical clustering
Lohoff *et al.* (2022)	seqFISH	50	57 536	24	None	Mouse embryos	Louvain clustering on top 50 PCs

**Figure 3.**
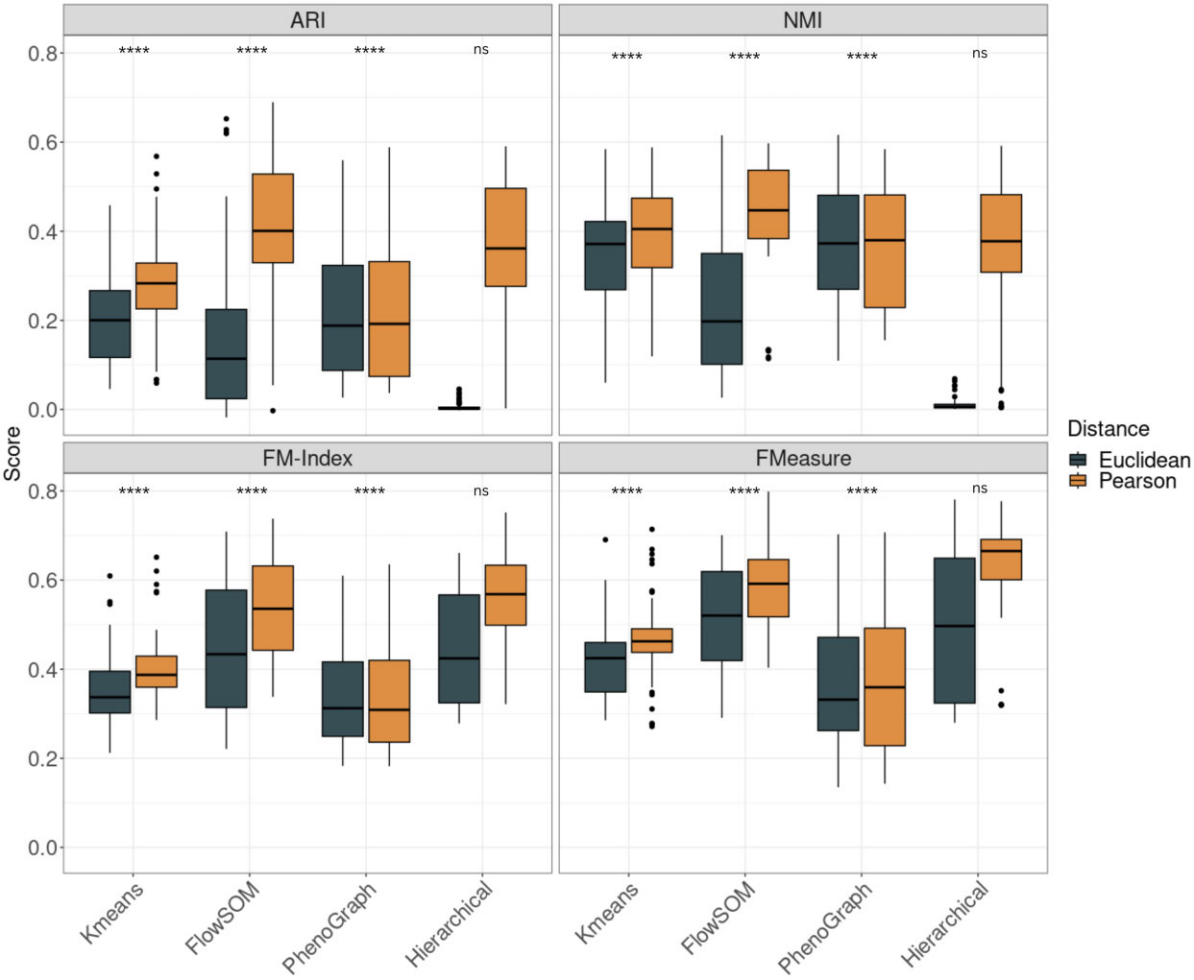
Boxplots of clustering performance of four clustering methods using Pearson correlation and Euclidean across four evaluation metrics (ARI, NMI, FM-index, and F-Measure). For FlowSOM, hierarchical clustering, and K-means, there is a statistically significant difference (****: P<.0001) in performance between Pearson and Euclidean using the Wilcoxon rank-sum test. This is not evident for PhenoGraph (ns: P>.05).

Next, we assessed if the performance differences between correlation and Euclidean distance are consistent across multiple clustering methods. We reconfigured PhenoGraph, FlowSOM, and K-means clustering to use correlation instead of Euclidean (Levine *et al.* 2015, Van Gassen *et al.* 2015). As previously, the 15 datasets were randomly subsetted to 20K cells five times and the performance scores were calculated for each subset. While there does not appear to be a benefit in clustering performance for PhenoGraph (P>0.05), the overall PhenoGraph performance for both distance measures is worse when compared to the other methods using correlation-based distances (Fig. 3 and Supplementary Fig. S2). For K-means, hierarchical clustering, and FlowSOM, we observe differences in the scores between Pearson correlation compared to Euclidean distance across all evaluation metrics (Fig. 3 and Supplementary Fig. S2).

### 3.2 Combining similarity metrics is beneficial

**Figure 4.**
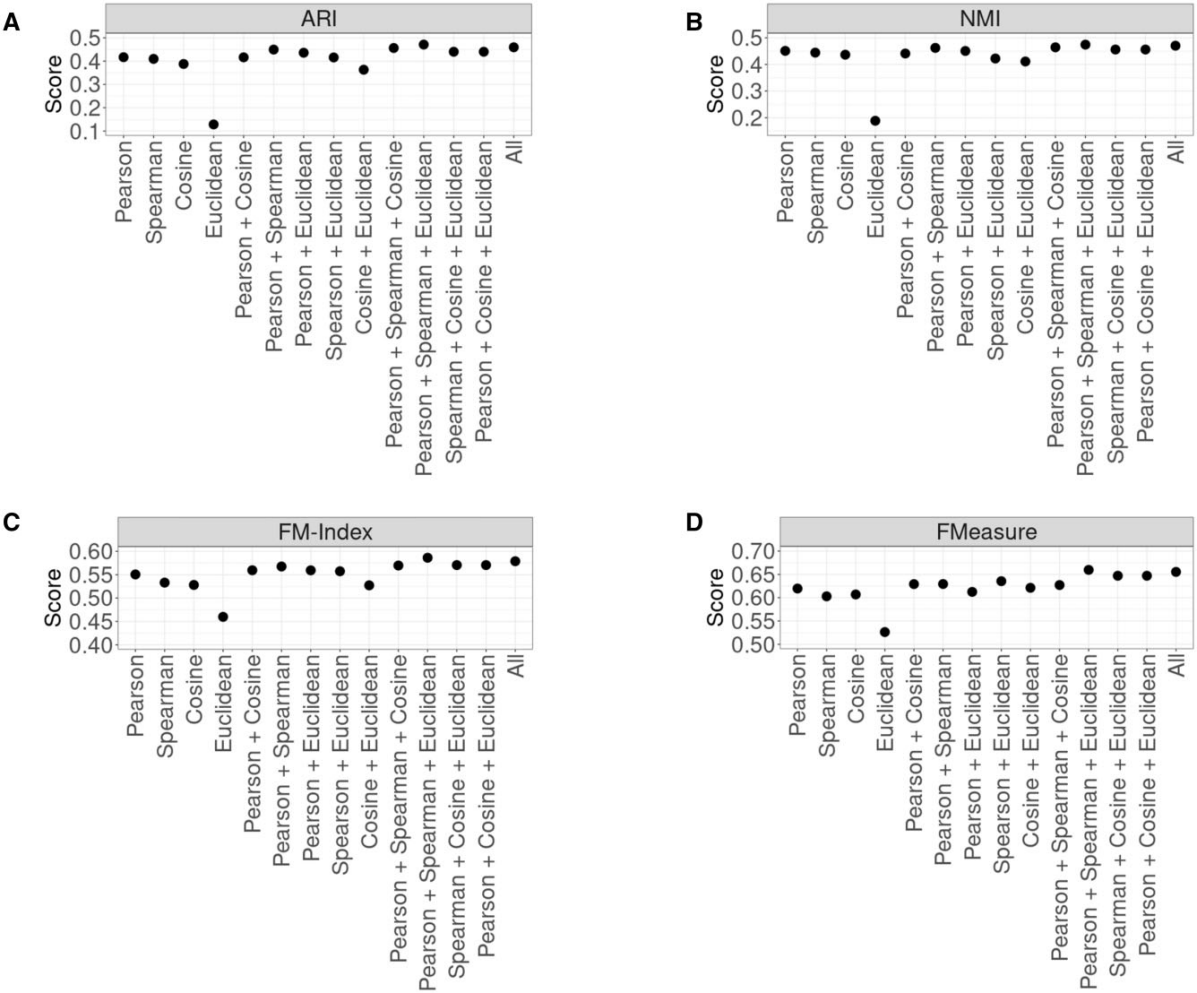
Scatter plots of average clustering performance of all combinations of Pearson, Cosine, Spearman, and Euclidean. (A) Scatter pot of the adjusted rand index. (B) Scatter plot of the normalized mutual information. (C) Scatter plot of the Fowlkes–Mallows index. (D) Scatter plot of the F Measure. Across all performance metrics, there is evidence that combining all distances provides a better signal for clustering.

Given the performance differences between the similarity metrics, we next assessed whether combining multiple metrics using strategies, such as multiview ensemble learning would further improve performance (Cao *et al.* 2020). To evaluate the efficacy of combining multiple distance metrics for clustering, we performed a comparison study combining various combinations of Pearson, Spearman, Cosine, and the Euclidean distance. All possible combinations of metrics were used to group the prototypes generated by the SOM algorithm and then the final scores were averaged across all datasets. Of all combinations, the combination of Pearson, Spearman, Cosine, and Euclidean consistently provided the best score in all four evaluation metrics (Fig. 4). The combination of Pearson, Spearman, and Cosine also provided strong results, which implies that the addition of Euclidean does not markedly improve the overall clustering performance.

### 3.3 FuseSOM combines SOMs with multiview ensemble learning of similarity metrics

**Figure 5.**
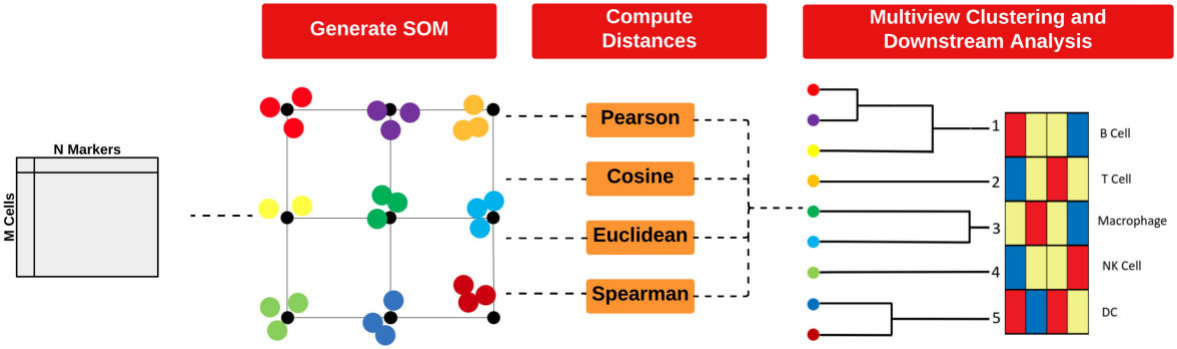
Overview of FuseSOM: this new scalable algorithm uses (i) a SOM to reduce the dimension of the data while preserving its topological structure; (ii) a multiview integration of various similarity metrics to capture all relevant signals; and (iii) hierarchical clustering to generate a clustering solution for further downstream analysis.

**Figure 6.**
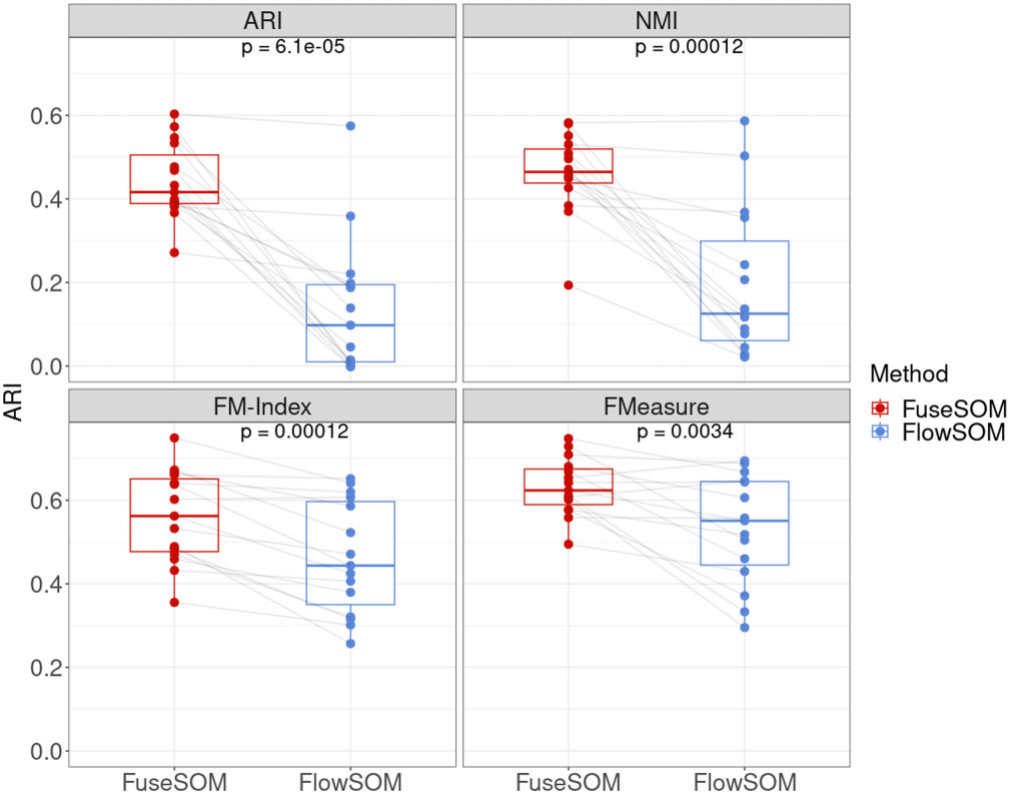
Paired boxplots for the average clustering performance across all datasets. For all four evaluation metrics, differences in performance are evaluated using the Wilcoxon rank-sum test.

Here, we introduce FuseSOM for the clustering of highly multiplexed imaging data. FuseSOM leverages all the ideas already discussed by combining similarity metrics with a SOM and multiview hierarchical clustering to define cell types robustly (Fig. 5). Compared to FlowSOM, which uses Euclidean distance by default, FuseSOM has superior performance in our stratified subsampling analysis framework (Fig. 6, P<0.05, and Supplementary Fig. S3), which demonstrates that a multiview ensemble of similarity metrics provides a more robust clustering. The performance gain is particularly evident when looking at ARI and NMI, with average differences in scores being 32% for ARI, 27% for NMI, 10% for FM-Index, and 9.0% for F-Measure.

**Figure 7.**
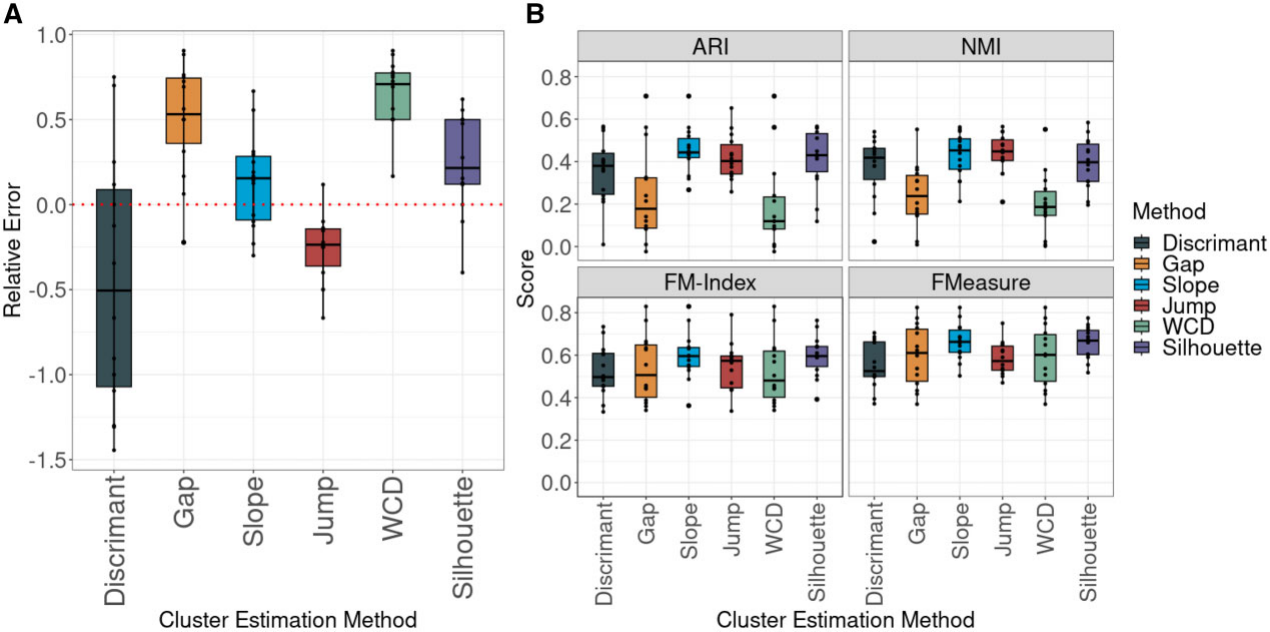
(A) Boxplots of cluster estimation performance for methods included in FuseSOM across all 15 datasets. The RE metric was used to gauge performance. The Jump and Discriminant methods are the top performers. The dashed line represents a RE of zero. In contrast, values above this line indicate an underestimation of the true number of clusters, and values below indicate an overestimation of the true number of clusters. (B) Boxplots of clustering performance based on the estimated number of clusters for methods included in FuseSOM. The Jump and Discriminant methods are top performers across all evaluation metrics.

Several methods for estimating the number of clusters have been implemented in the FuseSOM R package. The relative error (RE) between the predicted number of clusters and the number of clusters used in the corresponding manuscripts was used to assess the accuracy of the cluster estimation methods. The Jump and Discriminant method tends to overestimate the actual number of clusters while the others tend to underestimate the actual number of clusters (Fig. 7A). Next, FuseSOM was used to group cells in datasets using the number of groups estimated by each method. After estimating the number of clusters and then clustering, the REs and clustering scores were averaged in all datasets. When used for choosing the number of clusters, the Jump and Discriminant methods appear to have the highest average ARI and NMI, across all datasets (Fig. 7B). This demonstrates the complexity in evaluating the best metric for selecting the number of clusters.

To investigate the running time and memory usage of FuseSOM, we applied FuseSOM to the Hoch dataset (Hoch *et al.* 2022). This dataset contains 800K cells across 41 markers. Next, clustering was performed in increments of 50K cells starting from 100K cells to 400K cells (eight clustering solutions in total) to gauge how memory and running are affected by an increasing number of cells. Clustering was performed on an 11th Gen Intel^®^ Core™ i7-1165G7 @ 2.80 GHz with four cores and 32 GB of memory. FuseSOM performance was compared against FlowSOM and PhenoGraph. In terms of running time, FuseSOM scales well and is comparable to that of FlowSOM while being faster than PhenoGraph (Supplementary Fig. S4A). For memory usage, FuseSOM is more demanding than PhenoGraph, but less demanding than FlowSOM (Supplementary Fig. S4B). Doubling the size of the data requires twice as much memory for FuseSOM, while doubling the size of the data will require more than double the amount of memory for FlowSOM. The results show that FuseSOM provides a good balance between speed and memory consumption.

## 4 Discussion

In this work, we performed a comparative analysis of the performance of various similarity metrics for clustering highly multiplexed *in situ* imaging cytometry assays. Using multiple clustering methods across multiple similarity metrics, we demonstrate that the choice of similarity metric affects the clustering performance of highly multiplexed cytometry *in situ* imaging data with correlation-based metrics on average outperforming distance-based metrics. We then leveraged these findings to develop a novel multiview clustering algorithm called FuseSOM and demonstrated its ability to recover semi-supervised cell-type annotations across various datasets from differing imaging technologies with reasonable accuracy. Our results comprehensively demonstrate the impact of similarity metric choice on cell-type clustering in highly multiplexed imaging cytometry data and highlight the need to develop new best-practice clustering algorithms for these technologies.

While we have demonstrated that correlation metrics are often superior to distance metrics for multiplexed imaging data, we have not shown why this is the case. We do however demonstrate that this phenomenon is consistent across the imaging platforms. Our hypothesis is that distance-based metrics, such as Euclidean and Manhattan, are sensitive to the scaling of the data and therefore are susceptible to changes in the expression of markers across images or even different regions of the tissue imaged. However, correlation-based metrics, such as Pearson and Spearman, are scale-invariant and, therefore, could be less susceptible to changes in the expression of markers driven by technical artifacts. As correlation-based metrics only consider relative expression between markers, we suspect that this makes them more robust and, therefore, more accurate in capturing cell-type-specific expression trends in highly multiplexed *in situ* imaging cytometry data.

There are many analytical decisions and data properties that can impact the phenotyping of cells. In this manuscript, we have focused solely on the choice of distance metric used for clustering. To maintain this focus in our benchmarking study, for each dataset, we used the same cell segmentation, marker quantification, cross-image marker normalization, marker selection, and number of clusters that were used in the original manuscripts. It should be expected that each of these components would impact the clustering of cells. We hope that our collection of datasets will assist in future benchmarks of each of these components.

Choosing the number of clusters to use when clustering remains as much an art as a science. As such, selecting a suitable number of clusters should always be viewed in the context of the application. For example, to identify rare cell types in biological data, one might need to deeply cluster the data to find smaller populations of cells. When using quantitative approaches to select the number, like those we have implemented in FuseSOM, our results highlight that some methods tend to identify more clusters on average and others less. Furthermore, while many clustering algorithms require the number of clusters to be chosen before executing the algorithm, there are others, such as graph-based and density-based methods, that can estimate the number of clusters as part of the algorithm. However, often other parameters, which do need to be chosen, such as the size of the neighborhood in density approaches, can inadvertently affect the number of clusters. Ultimately, there is no golden rule when selecting the number of clusters. Therefore, we encourage a user to employ their domain expertise and to use a variety of the methods we have implemented to arrive at a sensible choice for the number of clusters.

## Supplementary Material

vbad141_Supplementary_Data

## Data Availability

Publicly available data were used for all evaluations. All data were downloaded as described in the originating manuscripts.

## Code availability

The *FuseSOM* R package is available on Bioconductor and is available under the GPL-3 license. All the codes for the analysis performed can be found at Github.
