# Supplementary material for: The impact of similarity metrics on cell-type clustering in highly multiplexed in situ imaging cytometry data
Source: Bioinform Adv. 2023 Oct 9;3(1):vbad141. doi: 10.1093/bioadv/vbad141 (PMC10625459; doi:10.1093/bioadv/vbad141)
Supplement: vbad141_Supplementary_Data [file vbad141_supplementary_data.pdf]

Supplementary data for "The impact of similarity metrics on cell-type clustering in highly multiplexed in situ imaging cytometry data"

Elijah Willie[1,2], Penyi Yang[1,2,3,4], Ellis Patrick[1,2,3,5]

[1]Sydney Precision Data Science Centre, The University of Sydney, Camperdown, NSW 2006, Australia
[2]School of Mathematics and Statistics, The University of Sydney, Camperdown, NSW 2006, Australia
[3]Laboratory of Data Discovery for Health Limited (D24H), Science Park, Hong Kong, China
[4]Computational Systems Biology Group, Children's Medical Research Institute, The University of Sydney, Westmead, NSW 2145, Australia
[5]Centre for Cancer Research, The Westmead Institute for Medical Research, The University of Sydney, Westmead, NSW 2145, Australia

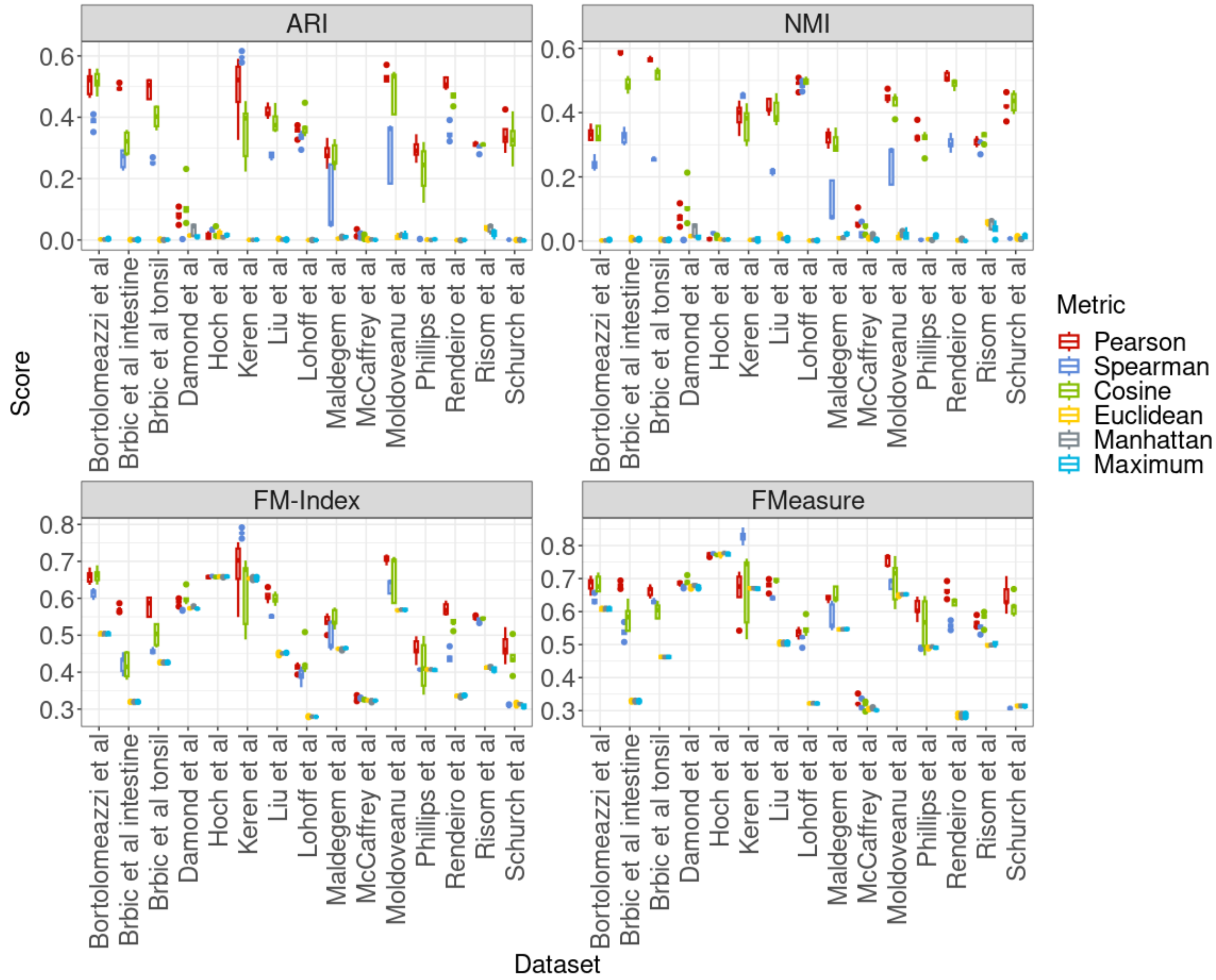

Supplementary Figure S1: Boxplots of benchmarking similarity metrics on agglomerative clustering of 15 multiplexed imaging datasets. Each dataset was subsetted to 20K cells five times, and the distribution of clustering scores was plotted. Correlation-based distances consistently have a higher average score compared to Euclidean-based distances.

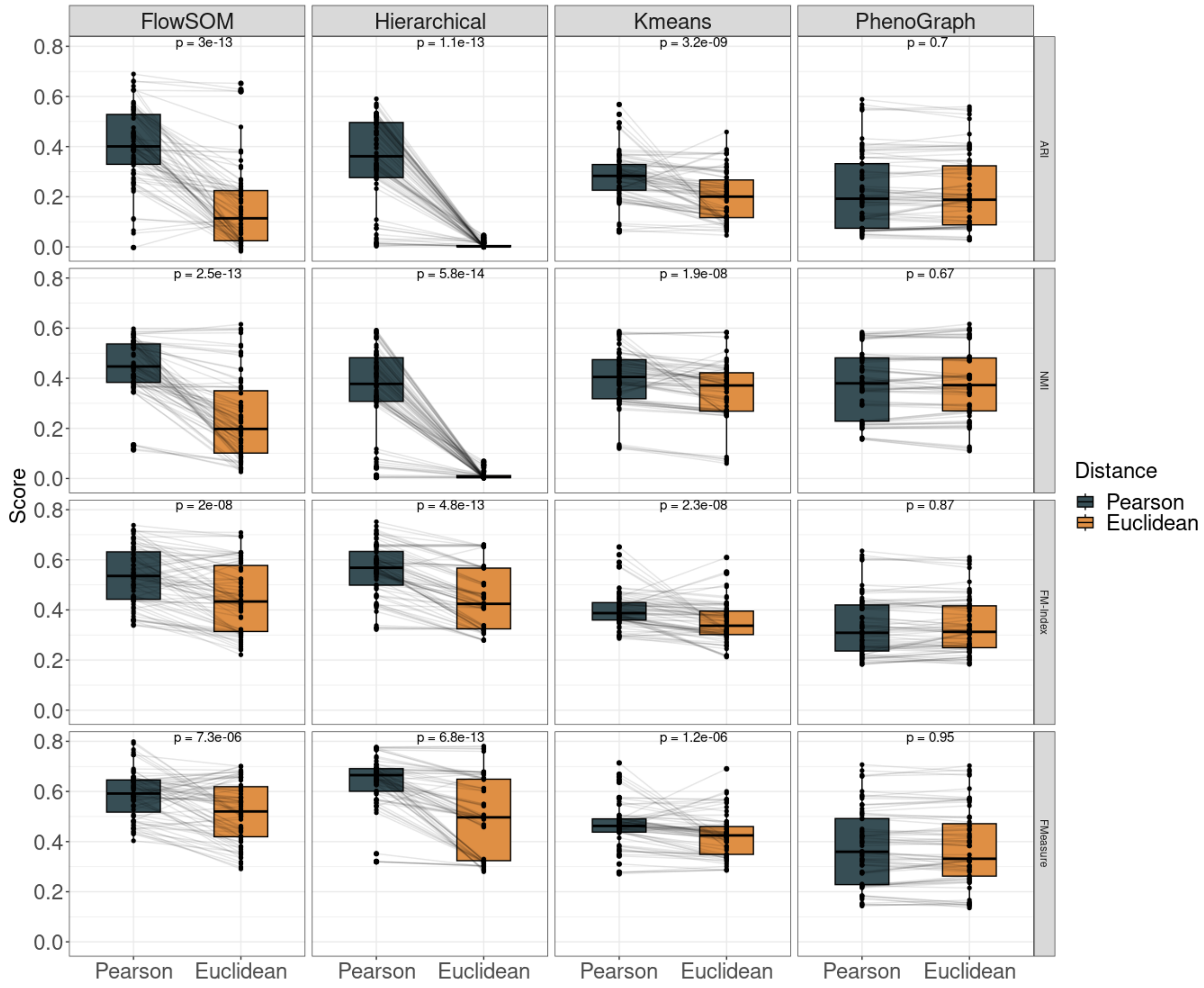

Supplementary Figure S2: Paired boxplots of clustering performance of four clustering methods using Pearson correlation and Euclidean across four evaluation metrics (ARI, NMI, FM-Index, and FMeasure). Actual range of p-values has been shown. For FlowSOM, Hierarchical clustering, and Kmeans, there is a statistically significant difference in performance between Pearson and Euclidean using the Wilcoxon rank-sum test. This is not evident for PhenoGraph

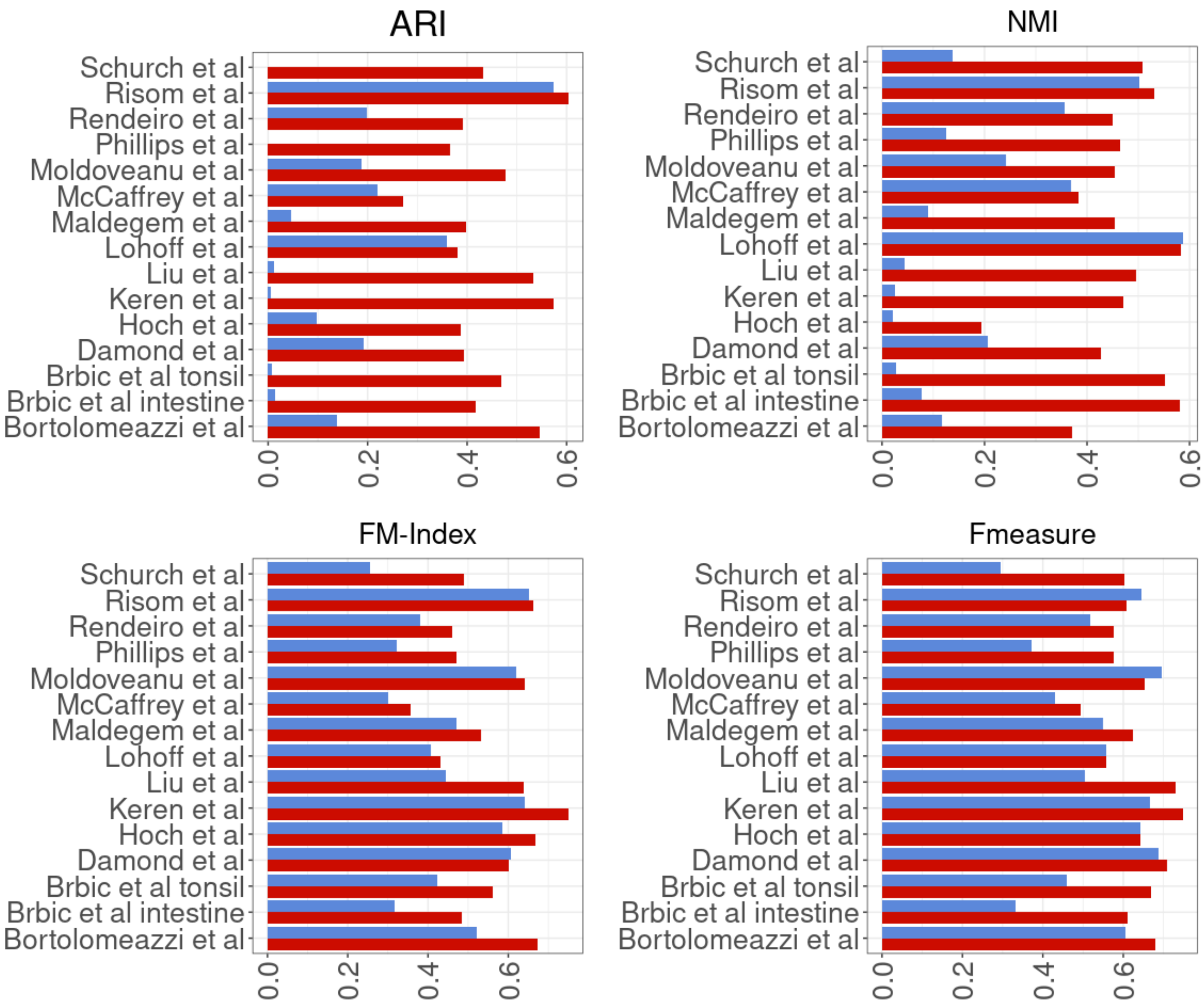

Supplementary Figure S3: Bar plots of average clustering performance for FuseSOM and FlowSOM showed for all 15 datasets across four performance metrics (ARI, NMI, FM-Index, and FMeasure). For a majority of the datasets, FuseSOM outperforms FlowSOM.

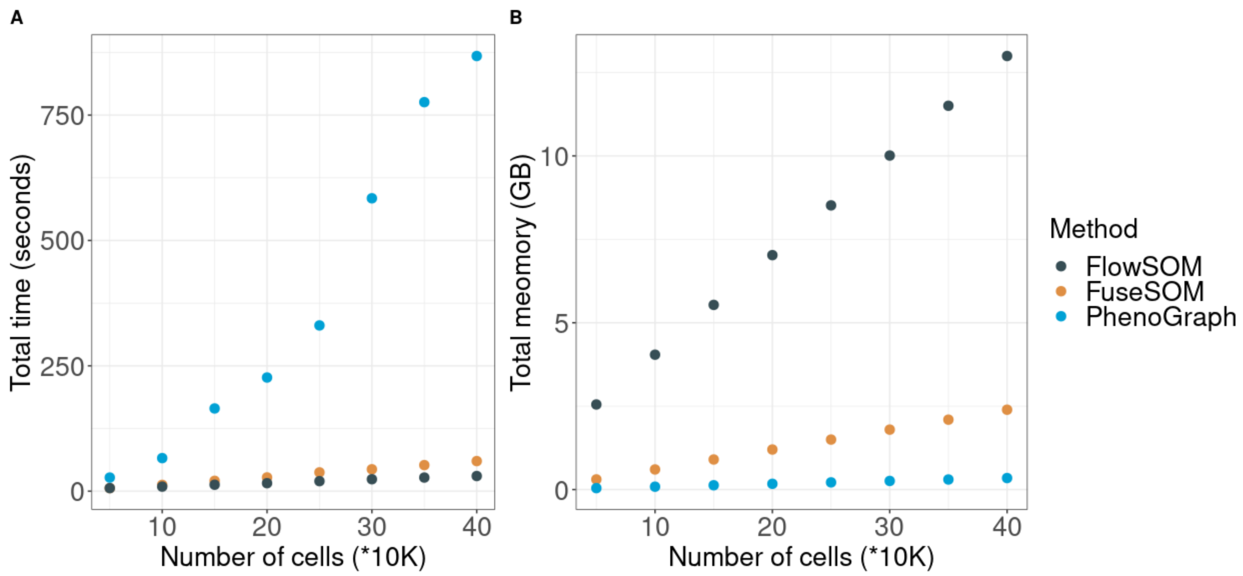

Supplementary Figure S4: (A) Scatter plot of total running time for FuseSOM, FlowSOM, and PhenoGraph across an increasing number of cells. (B) Scatter plot of total memory usage for FuseSOM, FlowSOM, and PhenoGraph across an increasing number of cells.

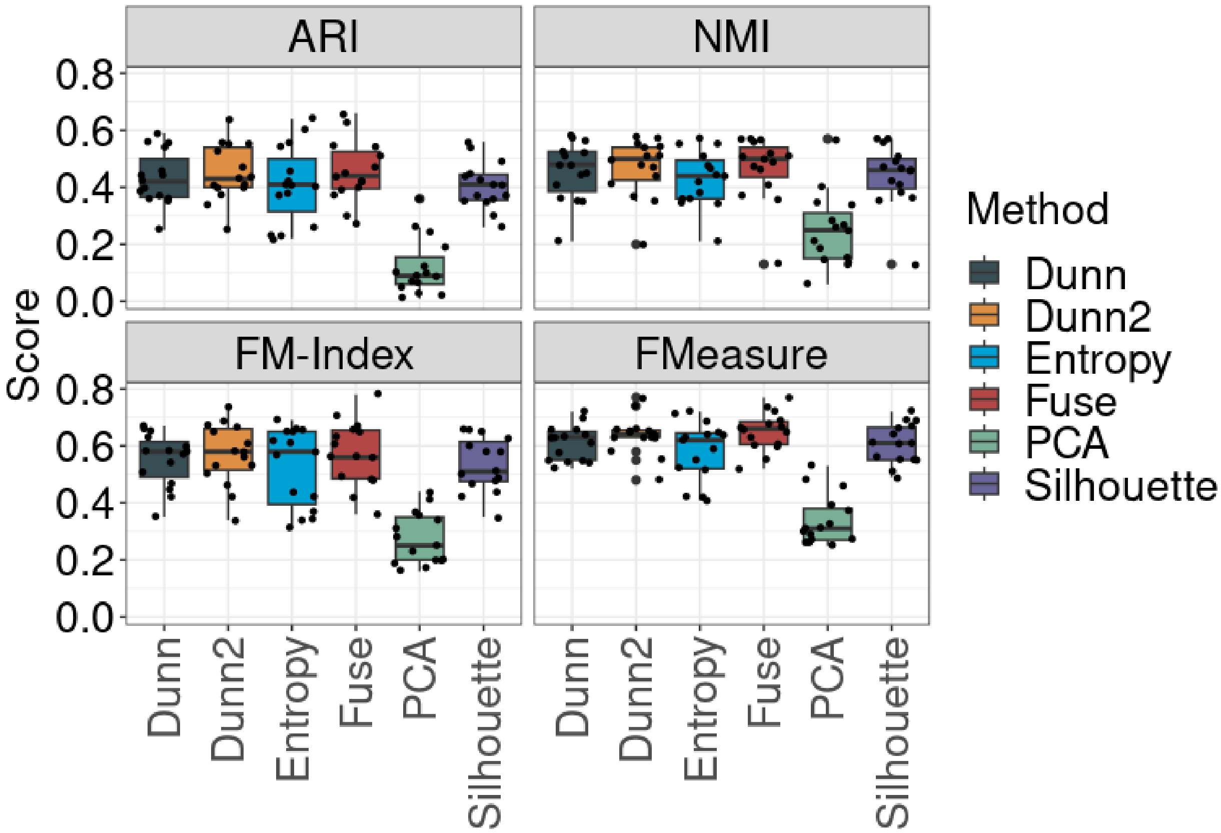

Supplementary Figure S5: Boxplots of clustering performance of different multiview weighting schemes. There appears to be no significant improvement in performance using other weighting schemes.

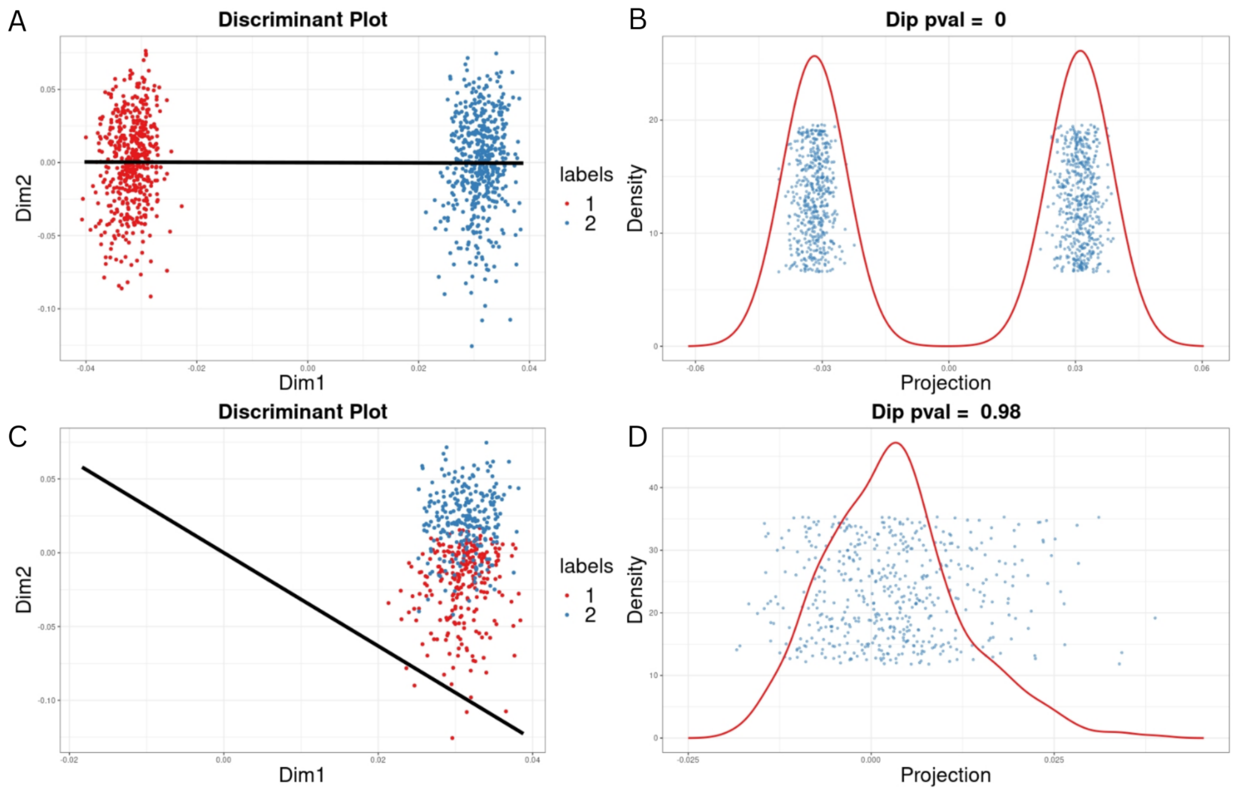

Supplementary Figure S6: An example of discriminant coordinates cluster number estimation. (A) Shows two classes well separated by a discriminant line in black. (B) Shows the Dip test applied to the projection of the two classes onto this discriminant line in (A). Note that a significant p-value ($p < 0.05$) is obtained for this case. (C) Shows two classes not well separated by a discriminant line in black. (D) Shows the Dip test applied to the projection of the two classes onto this discriminant line in (C). Note that a non-significant p-value ($p > 0.05$) is obtained for this case.
